# Mapping of a quantitative trait locus for resistance against infectious salmon anaemia in Atlantic salmon (*Salmo Salar*): comparing survival analysis with analysis on affected/resistant data

**DOI:** 10.1186/1471-2156-8-53

**Published:** 2007-08-14

**Authors:** Thomas Moen, Anna K Sonesson, Ben Hayes, Sigbjørn Lien, Hege Munck, Theo HE Meuwissen

**Affiliations:** 1 AKVAFORSK – Institute of Aquaculture Research, Ås, Norway; 2 CIGENE – Centre of Integrative Genetics, Ås, Norway; 3 Institute of Animal and Aquacultural Sciences, Norwegian University of Life Science, Ås, Norway

## Abstract

**Background:**

Infectious Salmon Anaemia (ISA) is a viral disease affecting farmed Atlantic salmon (*Salmo salar*) worldwide. The identification of Quantitative Trait Loci (QTL) affecting resistance to the disease could improve our understanding of the genetics underlying the trait and provide a means for Marker-Assisted Selection. We previously performed a genome scan on commercial Atlantic salmon families challenge tested for ISA resistance, identifying several putative QTL. In the present study, we set out to validate the strongest of these QTL in a larger family material coming from the same challenge test, and to determine the position of the QTL by interval mapping. We also wanted to explore different ways of performing QTL analysis within a survival analysis framework (i.e. using time-to-event data), and to compare results using survival analysis with results from analysis on the dichotomous trait 'affected/resistant'.

**Results:**

The QTL, located on Atlantic salmon linkage group 8 (following SALMAP notation), was confirmed in the new data set. Its most likely position was at a marker cluster containing markers BHMS130, BHMS170 and BHMS553. Significant segregation distortion was observed in the same region, but was shown to be unrelated to the QTL. A maximum likelihood procedure for identifying QTL, based on the Cox proportional hazard model, was developed. QTL mapping was also done using the Haley-Knott method (affected/resistant data), and within a variance-component framework (affected/resistant data and time-to-event data). In all cases, analysis using affected/resistant data gave stronger evidence for a QTL than did analysis using time-to-event data.

**Conclusion:**

A QTL for resistance to Infectious Salmon Anaemia in Atlantic salmon was validated in this study, and its more precise location on linkage group eight was determined. The QTL explained 6% of the phenotypic variation in resistance to the disease. The linkage group also displayed significant segregation distortion. Survival models proved in this case not to be more suitable than models based on the dichotomous trait 'affected/resistant' for analysing the data.

## Background

Infectious Salmon Anemia (ISA) is a viral disease causing substantial losses within the Atlantic salmon farming industry. The disease was first identified in Norway in 1984 [1], and has since emerged in Scotland [2], Canada [3], The United States [4], Chile [5], and the Faeroe Islands [6]. The causative agent belongs to the *Orthomyxoviridae *family of single-strand RNA viruses [7,8], as do the human influenza viruses. The mortalities of epidemics range from 15% to 100% [9]. ISA seems to cause disease only in farmed Atlantic salmon, though wild Atlantic salmon and other species of fish have been shown to be carriers of the virus [10]. Studies have shown that resistance to the disease has a genetic component, with narrow-sense heritability estimated to be 0.19 [11]. Resistance to ISA has been an objective of breeding for some salmon breeding programs since the mid 1990's, with resistance being defined as survival during ISA outbreaks and selection being done on the basis of family survival rates in artificial ISA challenge tests (family selection). If genetic markers associated to resistance against ISA could be found, they could be used to improve the selection process through Marker-Assisted Selection (MAS). In particular, MAS could provide a means for within-family selection of breeding candidates.

In an earlier study, a genome scan was performed to identify QTL for resistance to ISA in Atlantic salmon. The genome scan was performed on two full-sib families coming from the challenge tests of a major salmon breeding company, using Amplified Fragment Polymorphism (AFLP) markers and a multi-stage testing strategy [12]. Several putative QTL were identified in the study. At a later stage, a number of microsatellites were genotyped on the same material, and an AFLP/microsatellite linkage map was constructed from the data [13].

Challenge tests are often terminated before the test has gone to completion, i.e. before the survival curve has reached zero or levelled out. Often, one considers the dichotomous trait 'affected/resistant', usually meaning survival or not survival at the time the test was terminated. This trait, however, does not make use of the complete survival distribution. Survival models have been introduced to deal with time-to-event data in which individual records may be truncated, such as would occur in a challenge test that was terminated before completion. An often used group of survival models is the proportional hazard models, according to which the likelihood of an event is a function of a baseline hazard and an individual-specific term depending on the covariates of the individual. In the perhaps most frequently used proportional hazard model, the Cox model [14], the baseline hazard at any time interval is completely arbitrary.

Identified QTL should preferably be confirmed by validation of their segregation in additional families and populations [15]. In this study, we investigated the most promising QTL from the earlier study [12] in additional families, to validate the QTL, and to estimate its genomic position through interval mapping. In addition, we compared the effectiveness of survival models for analysing QTL data with that of models based on the trait 'affected/resistant'.

## Results

**Figure 1:**
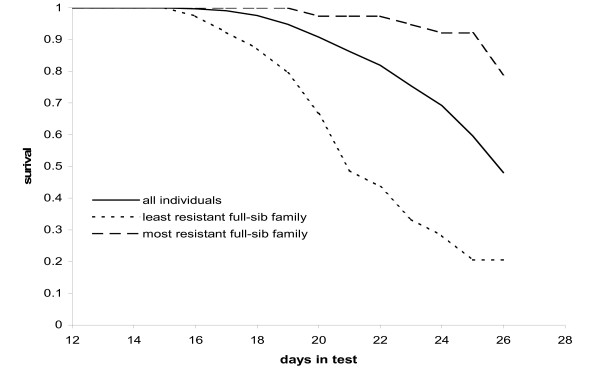
**Challenge test survival curves of animals used in the QTL experiment**. Survival = fraction of animals still alive; days-in-test = days passed since the beginning of the test.

The genetic material came from a challenge test performed by a major salmon breeding company, and was part of their routine calculation of breeding values. The challenge test was terminated when the overall survival rate was 0.48, and it followed a trajectory typical of an IPN infection (Figure 1). The survival rate within full- and half-sib families ranged from 0.18 to 0.78, with a standard deviation of 0.14 (Table 1). The survival curve (Figure 1) had not reached a plateau, indicating that further mortalities would be expected if the test had been allowed to continue. Consequently, the classification of fish as resistant or susceptible has a relative meaning in this study. The average weight of the subset of fish to be used in the present study was 31.4 g ± 6.4 g (SD), and there was a positive correlation between the trait affected/resistant and weight (Pearson correlation; r = 0.190, P < 0.0001).

**Table 1:** Survival rates within full-sib families

	**All families^a^**	**Genotyped families^b^**
Mean ± SD	0.46 ± 0.18	0.45 ± 0.14
Maximum	0.87	0.78
Minimum	0.03	0.18

^a^All families that were in the challenge test

^b^The families that were used for QTL mapping

**Figure 2:**
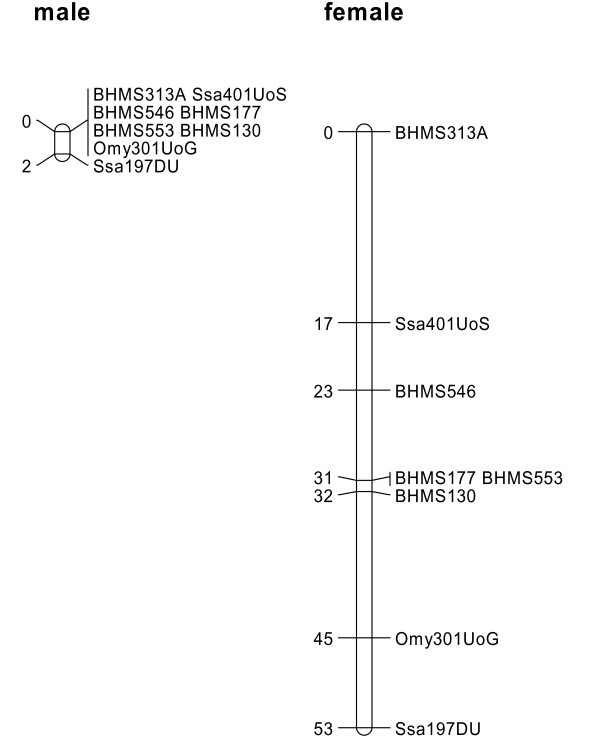
**Male- and female-specific maps of the Atlantic salmon linkage group 8**. The mapping unit is centi-Morgan (Kosambi mapping function).

The QTL to be confirmed was known to be located on linkage group 8 (LG8) following the SALMAP notation [12,13]. Eight microsatellite markers were therefore genotyped across this linkage group (Table 2). A linkage map was constructed from the data (Figure 2), and found to confirm the findings from Moen et al. [13] and from the SALMAP project [39] that the recombination rate in males is very low on this linkage group and that the few male recombination events that are observed occur within a limited region.

**Table 2:** Microsatellite markers used in this study

**Marker name**	**Alternative name**	**Accession number**	**Forward primer**	**Reverse primer**
Ssa197DU	-	U43694	TGAGTAGGGAGGCTTGTG	TGACATAACTCTTCTATGGC
Omy301UoG	-	-	ACTTAAGACTGGCAACCTT	CTACACGGCCTTCGGGTGAGA
BHMS130	Ssa12NVH	AF256663	AGTCAGAGACAACCCTCC	TGTCAGTCTGCTAAACACTG
BHMS177	Ssa22NVH	AF256673	GCTGTTCATCTGGCTGTG	TTCCATTTCCTCCCCCAG
BHMS553	Ssa87NVH	AF256732	CTGTAAACATCACAGGCG	CTCCACTAATAGTCTGAAGG
BHMS546	Ssa200NVH	AF256829	GGGACACTCATCTTGAATG	GGTAAGCATTTCACAGTAAG
Ssa401UoS	-	AJ402718	ACTGGTTGTTGCAGAGTTTGATGC	AAACATACCTGATTCCCGAACCAG
BHMS313A	Ssa99NVH	AF257052	TTCATGTGTGCGAGAGCG	AGAATGCAGTATTAGACTGG

**Figure 3:**
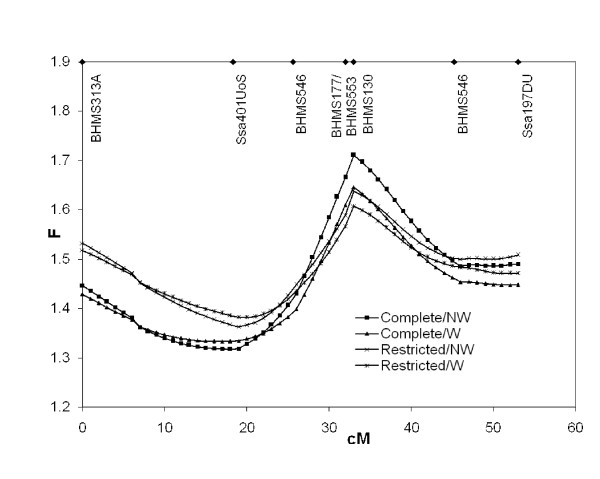
**Results from TPS-HK**. Complete = all families that were genotyped; Restricted = all families except those that were used in [12]; W = body weight included as covariate; NW = body weight not included as covariate; F = QTL Express F-statistic; cM = Kosambi centi-Morgan. Marker positions are indicated at the top. Permutation test significance levels can be found in Table 3.

The data were first analysed for QTL with the dichotomous trait Test-Period Survival and the Haley-Knott method for interval mapping (TPS-HK). The presence of the QTL on LG8 was confirmed in the new ('restricted') data set consisting of 15 Atlantic salmon full-sib families (Table 3, Figure 3). The QTL was more significant in the complete data set, consisting of the new families in addition to the ones used in Moen et al. [12]. The QTL was still significant after inclusion of weight as a covariate. The most likely QTL position was at 32 cM, although the 95% confidence interval for map position stretched across the entire linkage group. The QTL explained 6.0% of the phenotypic variance.

**Table 3:** Results from QTL mapping on the complete data set using the Haley-Knott method on the trait Test-Period Survival.

**Data set**	**Body weight as covariate**	**QTL position (cM)**	**LRT**
Restricted^a^	Yes	32	65.9*
Restricted^a^	No	32	67.5*
Complete^b^	Yes	32	71.9*
Complete^b^	No	32	74.7**

The significance levels of the Likelihood Ratio Test (LRT) statistic were computed by permutation testing.

*P < 0.05

**P < 0.01

^a^All families that were genotyped, except those that were used in the initial genome scan [12]

^b^All families that were genotyped

Analysis was also performed on individual parents, resulting in the detection of the QTL in 6 out of 44 parents based on nominal significance levels using either TPS-HK or TDS-Cox (Table 4) (4 of these were also chromosome-wise significant (P < 0.05)). Thus, 6/44 may serve as a lower estimate of the proportion of animals in the population segregating the QTL. When analysis was done on only these parents, the QTL was more significant (P < 0.001 chromosome-wise), the proportion of phenotypic variance explained by the QTL was larger (44.0%), but the 95% confidence interval for QTL position still covered the whole linkage group.

**Table 4:** Results from QTL mapping and test for Mendelian segregation on individual parents at the QTL peak position (32 cM).

**Family**	**Parent**	**Survival rate**	**TPS-HK (LRT)**	**TDS-Cox (LRT)**	**MENDEL (X^2^)**
1	sire	0.56	8.09**	6.65**	0.00
1	dam1	0.63	0.79	0.50	1.60
1	dam2	0.50	0.16	0.32	6.74**
2	sire	0.52	1.01	0.24	0.00
2	dam1	0.60	3.44	2.05	-
2	dam2	0.44	4.44*	2.22	0.23
3	sire	0.48	0.21	0.31	13.13**
3	dam1	0.53	0.00	0.09	0.90
3	dam2	0.44	8.03**	6.63*	3.79
4	sire	0.49	3.65	6.68**	3.10
4	dam1	0.49	0.15	0.65	1.26
5	sire	0.30	0.44	0.00	0.01
5	dam1	0.42	10.52**	6.73**	0.64
5	dam2	0.18	0.01	0.65	0.00
6	sire	0.41	3.80	3.63	0.01
6	dam1	0.33	3.78	2.91	0.10
6	dam2	0.49	1.35	0.75	1.26
7	sire	0.51	7.88**	6.30*	0.12
7	dam1	0.55	2.17	1.21	0.03
7	dam2	0.48	1.48	1.74	2.63
8	sire	0.51	0.15	0.12	2.25
8	dam1	0.58	1.15	2.12	0.24
8	dam2	0.39	2.18	2.23	0.03
9	sire	0.50	0.00	0.12	1.26
9	dam1	0.50	0.97	2.23	0.03
10	sire	0.56	1.21	1.71	0.03
10	dam1	0.56	0.03	0.00	0.03
11	sire	0.41	0.70	0.35	3.10
11	dam1	0.41	1.09	0.15	0.23
12	sire	0.56	0.01	0.00	0.00
12	dam1	0.78	1.09	0.93	0.68
12	dam2	0.35	1.28	1.08	1.32
13	sire	0.29	1.60	1.65	0.12
13	dam1	0.26	0.98	0.14	8.53**
13	dam2	0.33	0.05	1.08	0.03
14	sire	0.58	0.48	0.04	0.11
14	dam1	0.58	1.27	0.81	0.11
15	sire	0.55	0.77	0.56	2.92
15	dam1	0.74	0.71	0.68	0.00
15	dam2	0.36	0.19	0.01	5.77*
16	sire	0.39	0.22	0.27	0.00
16	dam1	0.39	0.00	0.16	4.24*
17	sire	0.28	0.00	0.01	0.23
17	dam1	0.28	0.20	0.11	0.03

The tests for QTL mapping are Likelihood Ratio Tests, distributed approximately as χ^2 ^with one degree of freedom. The test for Mendelian segregation is a Pearson's goodness-of-fit test, distributed approximately as χ^2 ^with one degree of freedom. Survival rate = survival rate among offspring of that parent; TDS = Test-Day Survival; TPS = Test-Period Survival. The Mendel tests were done at marker BHMS177, alternatively BHMS553 or BHMS130 when BHMS177 was not informative. The significance levels are nominal.

*P < 0.05

**P < 0.01

Some families had survival rates that were quite distant from 0.5, and thus contributed relatively little information to the data set. We therefore also analysed the data set without the families that had survival rates below 0.40 or above 0.60. With these 16 families excluded, the QTL was chromosome-wide significant at P < 0.001, and the QTL explained 9.0% of the phenotypic variance.

**Figure 4:**
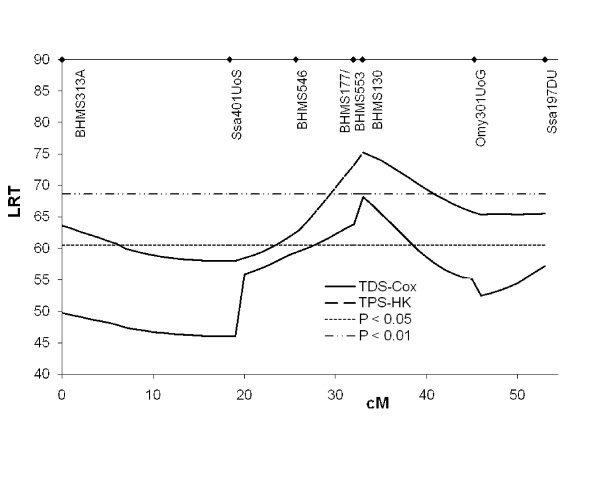
**Results from TDS-Cox**. Results from TPS-HK shown for reference, converted from an F-statistic to Likelihood-Ratio-Test (LRT) statistic by multiplying with the number of parameters fitted. cM = Kosambi centi-Morgan. Nominal significance levels are shown. Marker positions are indicated at the top.

We set out to test whether survival models could extract more information from our data than TPS-HK could. An interval mapping procedure was developed, based on the Cox proportional hazard model (termed Test-Day-Survival-Cox; TDS-Cox). The presence of the QTL was confirmed using this analysis method, albeit at a lower significance level when compared to TPS-HK (Table 4, Figure 4). The proportional hazard varied from 2.32 to 3.30 for offspring of parents that were segregating the QTL at P < 0.05. Hence, in these groups, animals having inherited the susceptibility allele from the parent in question were two to three times more likely to die at every day of the challenge test than animals having inherited the other allele.

**Figure 5:**
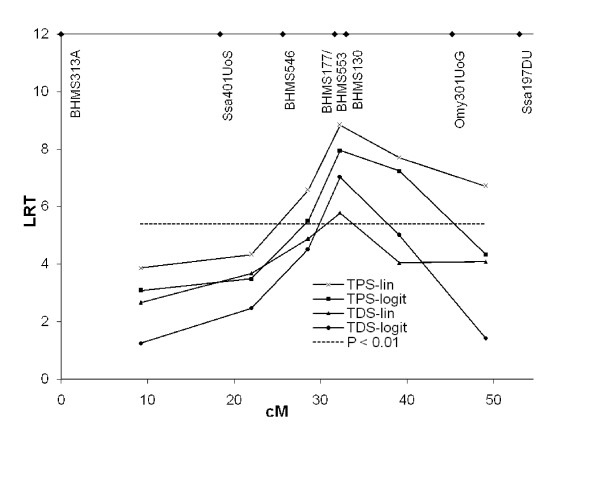
**Results from variance component analysis**. TPS = Test Period Survival (binary data model); TDS = Test Day Survival (survival model); LRT = Likelihood-Ratio-Test statistic; cM = Kosambi centi-Morgan; lin = linear model was used; logit; Generalised Linear Model with a logit link function was used. Nominal significance level is shown as broken line. Marker positions are indicated at the top.

Since the decreased evidence for QTL in TDS-Cox relative to TPS-HK was unexpected, we also analysed the data with both TDS and TPS within a variance component (VC) framework, where a more direct "within method" comparison between the use of TDS and TPS data is possible. By considering survival at individual test days as records and including Test Day as a fixed effect, the VC model used here has been shown to predict genetic effects on survival [16]. However, also in this case the analyses based on TPS (TPS-VC-lin and TPS-VC-logit) yielded higher significance levels than the survival analyses (TDS-VC-lin and TDS-VC-logit) (Figure 5). We also compared the analysis both with and without the use of a logit link function, which accounts for the binary nature of the traits. The comparisons indicated that the link function decreased evidence for a QTL with TPS, but increased evidence for a QTL with TDS (Figure 5). The QTL was found to explain 5.9% of the total phenotypic variance (TPS-VC-lin). Using nominal significance levels, the VC methods were found to give stronger evidence for QTL than did TPS-HK or TDS-Cox. We also tested for QTL for growth rate using Haley-Knott method, but did not find such a QTL.

Non-Mendelian segregation was observed on the linkage group to a greater extent than expected by chance (P < 0.01). The point of maximum non-Mendelian segregation coincided noticeably with the most likely QTL position (Table 5). However, there did not seem to be any relationship between the two findings, since i) there was a negative (though not statistically significant) correlation between the statistic for QTL and the statistic for non-Mendelian segregation for individual parents (r = -0.098; see also Table 4), and ii) the evidence for a QTL became more, not less, significant when parents displaying non-Mendelian segregation were excluded from analysis (result not shown). Non-Mendelian segregation was slightly more significant (p-value = 0.035) for female parents than for male parents (p-value = 0.067).

**Table 5:** P-values of tests for non-Mendelian segregation at different marker points.

**Marker**	**cM**	**P**
BHMS313A	0	0.076
Ssa401UoS	17	0.028
BHMS177/BHMS553	31	0.011
Omy301UoG	45	0.124
Ssa197DU	53	0.151

The p-values correspond to tests done across parents, at the respective marker points. The tests were not performed for three markers, since information contents were low for these markers compared to the other markers.

## Discussion

In this study, a putative QTL for ISA resistance in Atlantic salmon was tested in a larger data set coming from the same population. The effect of the QTL was confirmed in the new data set, and in all tested statistical analyses, strongly indicating that this is a true QTL and not a false positive.

The QTL investigated here was originally found in a genome scan for ISA resistance. However, the QTL was not among the set of QTL reported earlier [12], as the QTL investigated here was excluded from the final analysis stage in the previous study due to significant non-Mendelian segregation. Non-Mendelian segregation was a concern in this earlier study because the markers used were random markers not connected into a linkage map, meaning that non-Mendelian segregation could be a sign of genotyping error. At a later stage, however, addition of microsatellites to the marker set and the construction of a linkage map [13] made it evident that the observed non-Mendelian segregation was consistent across the linkage group investigated in the present study (ruling out genotyping error as a cause). Since this LG contained by far the most and strongest associations with the trait, it was chosen as the most interesting one for a follow-up study (markers acactg299, aggcat376, actcta476, aacctg366, and aggcat346, on top of Table 3 in Moen *et al*. [12], all belong to this LG).

In addition to confirming the QTL in a larger data set and to refine its map position, this study investigated whether survival models would be more suitable for analysing challenge test data than binary models. We used the P-values of the test-statistics as measures of the power to detect a QTL for various methods, since the test with the lowest P-value is still able to detect a QTL at this P-value whereas the other tests are not. The translation of the test-statistics into nominal P-values made it possible to compare methods across the various test-statistics. Our *a priori *hypothesis was that survival models would be able to extract more information from the data, since these models take the full distribution of the data into account. Contrary to our expectations, however, the best performing analysis methods were the ones based on the dichotomous TPS trait. A more in-depth investigation of the merits of survival models versus conventional methods in relation to survival analysis would require simulated data, and is outside the scope of the study. Within the present context, however, we conclude that survival models were not more effective at detecting QTL than were models based on the dichotomous TPS data, even though the data were in this case *a priori *believed to be more suitable for survival analysis (the overall survival rate was close to 50%, but there was a large variation in survival rates between families). It is possible that there were aspects of our data that disfavoured survival analysis, aspects that may not be relevant in other data sets. For example, for the VC methods, the sample size may not have been large enough for the estimation of the large number of effects implicit in the survival model. For the Cox method, the assumption of a limited number of ties may possibly have be unrealistic given the data, although it must be said that another implementation, allowing for a large number of ties according to [17,18], was also tested and found to give lesser evidence for a QTL than the method described in this paper (data not shown). It is also possible that there may be underlying biological reasons for the differences in 'performance' of the methods. For example, resistance to the disease at the early stages of the challenge test may have been determined by immunological factors other than those determining resistance at the later stages, or mortalities at early stages may have been due to the fish being generally weakened by the challenge test rather than dying from the disease itself. If either of these hypotheses were true, the correlation between QTL alleles and resistance would have been weaker at the earlier stages of the challenge test than at later stages. The survival models, by putting more emphasis on earlier deaths relative to later deaths than to the other models, would consequently give lower test statistics. In such a situation, neither of the models could be said to give 'truer' results than the other, since the interpretation of the results would depend upon what one was searching for (e.g. a QTL for survival in a challenge test or a QTL that is directly involved in resistance to the virus), but there would be a potential for learning more about the details underlying resistance by combining different methods.

There is a tendency in the data for families with survival rates distant from 0.5 to present less evidence for a QTL. This is likely to be due to these families having less power to detect QTL. At the beginning of the study, we judged that also families with survival rates most distant from 0.5 would be sufficiently informative for QTL analysis, in particular since we believed survival analysis would be able to extract more information from the data (at least for the families with survival rates larger than 0.5). In addition, some full-sib groups with survival rates distant from 0.5 were included because they had half-sib relationships to groups with survival rates close to 0.5 (and because complete half-sib groups were wanted). In retrospect, we may have decreased to power of the experiment to detect QTL by including some families with survival rates too far from 0.5.

A QTL for growth rate has earlier been found on the linkage group investigated here [19]. Since body size could be correlated with disease resistance traits, we included body size as a covariate in the initial analysis for QTL (TPS-HK). We did this as a precaution, and in spite of the fact that the genetic correlation between ISA resistance and growth rate has been shown to be close to zero (r = -0.032; Sissel Kjøglum, Aqua Gen, pers. comm.). In the present study, a positive and highly significant correlation was found, but it is important to note that body weight was measured at the end of the challenge test, meaning that the detected correlation may be due to larger fish being more resistant and/or more resistant fish being heavier because they are also older. After the QTL had been confirmed also when body weight had been corrected for, and after we had found no QTL for body weight on the linkage group, we chose to exclude the covariate from the remaining analyses, since we saw that it, as measured here, was confounded with survival.

Non-Mendelian segregation turned out to be a general trend on the linkage group, with many parents displaying highly significant segregation distortion. There are at least two possible explanations for the observed non-Mendelian segregation: A gene affecting early survival could cause differences in early survival between offspring having inherited one or the other allele at a linked marker, resulting in apparent segregation distortion at the time of challenge testing (eight months after first feeding). However, since at least 50% of eggs would be expected to be successfully fertilised and yield offspring that are still alive at eight months [20], the effect of this gene (or genes) would have to explain a very large fraction of variation in survival. Alleles having such a large negative effect on survival would be expected to have very low frequencies in the population, making this hypothesis unlikely. Another possibility is meiotic drive, the preferential formation of gametes having one allele instead of the other by molecular mechanisms. Meiotic drive has been reported and studied in diverse organisms (reviewed in [21], though to our knowledge not in salmonids). Segregation distortion at a level greater than that expected by chance has also been noted in Brown Trout [22].

In this study we have confirmed the presence of a QTL for ISA resistance on linkage group 8 of the Atlantic salmon. The QTL segregates in a commercial population of salmon that originates from a broad selection of Norwegian rivers [23]. The QTL explains 6–9% of the phenotypic variance in the population (6% in the complete data set, 9% when the least informative families were excluded). However, analysis performed on the parents that were individually significant for the QTL showed that the effect of the QTL was very large in those families. Also, given the moderate heritability of the trait (0.19 according to [11], the QTL explains a significant fraction of the additive genetic variance of the trait (32 – 47%). The QTL could thus be used for Marker-Assisted Selection on an economically important trait. A Transmission Disequilibrium Test (TDT) (taking into account affected and resistant animals, as well as multiple loci and alleles, as laid out in [24]) and combined linkage disequilibrium/linkage analysis (LDLA; [25]) showed that the QTL markers are not in population-wide association to ISA resistance (results not shown). This means that the QTL markers are likely to be some distance away from the functional polymorphism(s), as would be expected. Fine-mapping could be used to identify markers in linkage-disequilibrium with the functional polymorphism, or even the functional polymorphism itself. With new developments in salmonid genomics, such as a high-density Single Nucleotide Polymorphisms (SNP) map currently being developed by the cGRASP consortium [26], such ambitions are becoming realistic also in non-model species such as the Atlantic salmon.

## Conclusion

In this study, we have validated a QTL for resistance to Infectious Salmon Anaemia on Atlantic salmon linkage group 8. Significant non-Mendelian segregation was detected in the same genomic region, but was not related to the QTL. Comparing different methodologies for the analysis of the survival data, we found that, in our case, survival analysis was not more powerful than analysis on the dichotomous trait affected/resistant.

## Methods

### Genetic material, challenge test, and traits

The genetic material used came from a challenge test performed by VESO Vikan Ltd. (Namsos, Norway) on behalf of the Norwegian salmon breeding company Aqua Gen Ltd. (Trondheim, Norway). The Aqua Gen breeding program is large family-based breeding programme with inbreeding control, founded in the 1970's from a base consisting of salmon from 41 different Norwegian rivers. The accepted level of increase in the inbreeding coefficient of the Aqua Gen population is set to 0.5% per generation (Sissel Kjøglum, Aqua Gen, personal communication). Challenge tests for ISA are performed on a routine basis in connection with the Aqua Gen breeding programme, resistance to ISA being part of the breeding goal of Aqua Gen since 1994. In 2000, eight months after first feeding, fish from the 1999 year class of Aqua Gen were transported to VESO Vikan, put into a single tank with 12°C water temperature, acclimatized for nine days, and then intraperitoneally injected with infectious material (ISA strain Glesvaer/2/90). Dead fish were collected every day, and the test was terminated when approximately 50% of the fish overall had died, which was at test day 27. Body weight was measured at the end of the test, implicating that fish were of different ages when they were measured.

The dichotomous trait Test-Period Survival (TPS) was defined as the survival/not survival status of animals at the end of the test period (i.e. day 27). The trait Test-Day Survival (TDS) was defined as survival/not survival status at individual test intervals (i.e. days).

Among the fish that died before the end of the test, 90 (randomly selected) were autopsied to confirm ISA infection. 10% of the animals were tested for cross-infection by other pathogens using bacteriological tests (cross-infection was not detected). Further details on the challenge test can be found in [12].

Within the breeding nucleus, sires were at the time mated to two dams each, producing paternal half-sib family groups each consisting of two full-sib families. Out of a larger number of families tested in the challenge test, 10 such half-sib groups (i.e. 20 full-sib families), and an additional 7 full-sib families were genotyped and used in the present study. The selection of families to be genotyped was based on the distribution of affected versus resistant animals within families, a 50:50 distribution considered optimal. 40 fish from each full-sib family were challenged. Since the study aimed at testing the reproducibility of a putative QTL identified in an earlier study [12], the analysis was done on both i) all genotyped families ('complete data set') and ii) all genotyped families except the two full-sib families ones that were investigated in the earlier study ('restricted data set'). The complete data set consisted of 1053 individuals.

### Microsatellite genotyping

One linkage group (LG) was targeted for investigation in this follow-up study, corresponding to LG1 in [13] and LG8 on the SALMAP Atlantic salmon map (B. Høyheim, unpublished data [39]). Eight microsatellite markers from this linkage group were selected (Table 2). DNA was extracted from muscle tissue, using the DNAeasy Tissue Kit (96 well format) from QIAGEN (Venlo, The Netherlands). Microsatellite PCR was performed in 10 μl reactions containing 1× PCR-buffer with 1.5 mM MgCl_2_, 0.5 U Taq Gold polymerase (Applied Biosystems, Foster City, CA), 200 μM of each dNTP, 5% dimethylsulphoxide (DMSO), 250 nM of each primer, and 10 ng template. An annealing temperature of 52°C was used on all microsatellites. The electrophoresis was done on a 3730 DNA sequencer from Applied Biosystems, and genotypes were analyzed using GeneMapper 3.0 software (Applied Biosystems, Foster City, CA).

### Linkage analysis

Linkage analysis was done using the program Joinmap 3.0 [27]. For each full-sib family, the data was first split into two sets, containing data on alleles inherited from sires and dams, respectively. Data from all sires were pooled, as were the data from all dams, using the "Combine Groups for Map Integration" command of Joinmap 3.0. Sex-specific maps were then made. The default settings of the program were used for map construction. Since the male recombination rate is close to zero on this linkage group [13], the female map was used for QTL interval mapping. Following construction of the map, the data was checked for double recombinants (indicative of genotype error) using a Visual Basic for Applications (VBA) script running from Microsoft Excel (T. Moen, unpublished). When double recombinants were found, genotypes were checked, re-genotyped, and excluded if ambiguous. After checking for double recombinants and correction of data, the data set contained no double recombinants, supporting earlier findings of complete interference in salmonids [31-34].

### Test for non-Mendelian segregation

At each marker, individual parents were tested for a 1:1 segregation of alleles, using a χ^2 ^goodness-of-fit test. An overall test statistic was calculated as the sum of χ^2 ^test statistics for individual parents. The test was implemented through a Visual Basic-for-Excel script.

### QTL mapping

#### Interval mapping using Haley-Knott regression on test-period survival (TPS-HK)

Interval mapping on TPS was performed using the "Half-sib Analysis" option of QTL Express [28], based on the method of KNOTT *et al*. [29]. A one-QTL model was used, and analysis was performed at every 1 cM. In order to include contributions from both sires and dams, every record was duplicated, and in the duplicates the denomination of parents as sires or dams were switched. Analysis was done with and without body weight as a covariate. Permutation testing and bootstrapping, as implemented in QTL Express, were used to determine chromosome-wise significance levels of the test statistic and confidence intervals for QTL position, respectively. In both cases, the number of iterations was 500. The proportion of phenotypic variance was calculated using the formula 4(1-MS_full_/MS_reduced_), where MS is the residual mean square from the regression analysis [28]. The proportion of additive genetic variance explained by the QTL was found by dividing the proportion of phenotypic variance by the estimated heritability h^2 ^= 0.19 [11].

#### Interval mapping using the Cox proportional hazard model on test-period survival (TDS-Cox)

A method based on the Cox proportional hazard model was developed for interval mapping of TDS. In the Cox model [14], the hazard function of an individual with covariate vector **x **is the product of an arbitrary (nonparametric) baseline hazard function λ_0 _and a parametric function **e^x'β ^**of **x**. For our application, we used a version of the Cox partial likelihood that accounts for a small number of ties (more than one failure occurring within the same time interval) according to Peto and Peto [30,18]. A single-QTL model was assumed, with the QTL (Q) being separated by the nearest flanking markers A and B by map distances r_A _and r_B_, respectively. At every 1 cM, the maximum log likelihood of the data was calculated under the null hypothesis of no QTL affecting survival and under the alternative hypothesis of a QTL affecting survival during challenge. The log likelihood function was

LogL=∑T∈{unc.}[(∑i∈D(T)xiβ)−dT(log⁡∑j∈R(T)exjβ)]
 MathType@MTEF@5@5@+=feaafiart1ev1aaatCvAUfKttLearuWrP9MDH5MBPbIqV92AaeXatLxBI9gBaebbnrfifHhDYfgasaacH8akY=wiFfYdH8Gipec8Eeeu0xXdbba9frFj0=OqFfea0dXdd9vqai=hGuQ8kuc9pgc9s8qqaq=dirpe0xb9q8qiLsFr0=vr0=vr0dc8meaabaqaciaacaGaaeqabaqabeGadaaakeaacqWGmbatcqWGVbWBcqWGNbWzcqWGmbatcqGH9aqpdaaeqbqaaiabcUfaBjabcIcaOmaaqafabaGaemiEaG3aaSbaaSqaaiabdMgaPbqabaacciGccqWFYoGyaSqaaiabdMgaPjabgIGiolabdseaejabcIcaOiabdsfaujabcMcaPaqab0GaeyyeIuoaaSqaaiabdsfaujabgIGiolabcUha7jabdwha1jabd6gaUjabdogaJjabc6caUiabc2ha9bqab0GaeyyeIuoakiabcMcaPiabgkHiTiabdsgaKnaaBaaaleaacqWGubavaeqaaOGaeiikaGIagiiBaWMaei4Ba8Maei4zaC2aaabuaeaacqWGLbqzdaahaaWcbeqaaiabdIha4naaBaaameaacqWGQbGAaeqaaSGae8NSdigaaaqaaiabdQgaQjabgIGiolabdkfasjabcIcaOiabdsfaujabcMcaPaqab0GaeyyeIuoakiabcMcaPiabc2faDbaa@6A2B@

where {*unc*.} is the set of uncensored time intervals T, i.e. time intervals (days) before the challenge test was terminated; *D*(*T*) is the set of offspring that died within time interval *T*; *d*_*T *_is the number of offspring in *D*(*T*); *R*(*T*) is the set of offspring at risk at the beginning of time interval *T*; *x*_*i *_is *P*_*i*_(*Q*_1_) - *P*_*i*_(*Q*_2_); *P*_*i*_(*Q*_1_) and *P*_*i*_(*Q*_2_) are the probabilities of animal *i *having inherited one or the other QTL allele from the parent in question; *β *is a regression coefficient. For the calculation of *P*_*i*_(*Q*_1_) and *P*_*i*_(*Q*_2_), complete interference was assumed, a realistic assumption in salmonids [31-34]. Thus, if QTL allele Q_1 _was assumed to be in coupling phase with marker alleles A_1 _and B_1_, the probability of Q_1 _being inherited by animals having marker genotypes A_1_B_1_, A_1_B_2_, A_2_B_1_, or A_2_B_2 _was 1, rBrA+rB
 MathType@MTEF@5@5@+=feaafiart1ev1aaatCvAUfKttLearuWrP9MDH5MBPbIqV92AaeXatLxBI9gBaebbnrfifHhDYfgasaacH8akY=wiFfYdH8Gipec8Eeeu0xXdbba9frFj0=OqFfea0dXdd9vqai=hGuQ8kuc9pgc9s8qqaq=dirpe0xb9q8qiLsFr0=vr0=vr0dc8meaabaqaciaacaGaaeqabaqabeGadaaakeaadaWcaaqaaiabdkhaYnaaBaaaleaacqWGcbGqaeqaaaGcbaGaemOCai3aaSbaaSqaaiabdgeabbqabaGccqGHRaWkcqWGYbGCdaWgaaWcbaGaemOqaieabeaaaaaaaa@35A2@, rArA+rB
 MathType@MTEF@5@5@+=feaafiart1ev1aaatCvAUfKttLearuWrP9MDH5MBPbIqV92AaeXatLxBI9gBaebbnrfifHhDYfgasaacH8akY=wiFfYdH8Gipec8Eeeu0xXdbba9frFj0=OqFfea0dXdd9vqai=hGuQ8kuc9pgc9s8qqaq=dirpe0xb9q8qiLsFr0=vr0=vr0dc8meaabaqaciaacaGaaeqabaqabeGadaaakeaadaWcaaqaaiabdkhaYnaaBaaaleaacqWGbbqqaeqaaaGcbaGaemOCai3aaSbaaSqaaiabdgeabbqabaGccqGHRaWkcqWGYbGCdaWgaaWcbaGaemOqaieabeaaaaaaaa@35A0@, and 0, respectively. The likelihood ratio test (LRT) statistic was

LRT=∑i=1N2[LogL^(H1)−LogL^(H0)]
 MathType@MTEF@5@5@+=feaafiart1ev1aaatCvAUfKttLearuWrP9MDH5MBPbIqV92AaeXatLxBI9gBaebbnrfifHhDYfgasaacH8akY=wiFfYdH8Gipec8Eeeu0xXdbba9frFj0=OqFfea0dXdd9vqai=hGuQ8kuc9pgc9s8qqaq=dirpe0xb9q8qiLsFr0=vr0=vr0dc8meaabaqaciaacaGaaeqabaqabeGadaaakeaacqWGmbatcqWGsbGucqWGubavcqGH9aqpdaaeWbqaaiabikdaYmaadmaabaGaemitaWKaem4Ba8Maem4zaCMafmitaWKbaKaacqGGOaakcqWGibasdaWgaaWcbaGaeGymaedabeaakiabcMcaPiabgkHiTiabdYeamjabd+gaVjabdEgaNjqbdYeamzaajaGaeiikaGIaemisaG0aaSbaaSqaaiabicdaWaqabaGccqGGPaqkaiaawUfacaGLDbaaaSqaaiabdMgaPjabg2da9iabigdaXaqaaiabd6eaobqdcqGHris5aaaa@4DBA@

Where *N *is the number of parents, and LogL^(H0)
 MathType@MTEF@5@5@+=feaafiart1ev1aaatCvAUfKttLearuWrP9MDH5MBPbIqV92AaeXatLxBI9gBaebbnrfifHhDYfgasaacH8akY=wiFfYdH8Gipec8Eeeu0xXdbba9frFj0=OqFfea0dXdd9vqai=hGuQ8kuc9pgc9s8qqaq=dirpe0xb9q8qiLsFr0=vr0=vr0dc8meaabaqaciaacaGaaeqabaqabeGadaaakeaacqWGmbatcqWGVbWBcqWGNbWzcuWGmbatgaqcaiabcIcaOiabdIeainaaBaaaleaacqaIWaamaeqaaOGaeiykaKcaaa@35AB@ and LogL^(H1)
 MathType@MTEF@5@5@+=feaafiart1ev1aaatCvAUfKttLearuWrP9MDH5MBPbIqV92AaeXatLxBI9gBaebbnrfifHhDYfgasaacH8akY=wiFfYdH8Gipec8Eeeu0xXdbba9frFj0=OqFfea0dXdd9vqai=hGuQ8kuc9pgc9s8qqaq=dirpe0xb9q8qiLsFr0=vr0=vr0dc8meaabaqaciaacaGaaeqabaqabeGadaaakeaacqWGmbatcqWGVbWBcqWGNbWzcuWGmbatgaqcaiabcIcaOiabdIeainaaBaaaleaacqaIXaqmaeqaaOGaeiykaKcaaa@35AD@ are the maximum log likelihoods under the null- and alternative hypotheses, respectively. LogL^(H1)
 MathType@MTEF@5@5@+=feaafiart1ev1aaatCvAUfKttLearuWrP9MDH5MBPbIqV92AaeXatLxBI9gBaebbnrfifHhDYfgasaacH8akY=wiFfYdH8Gipec8Eeeu0xXdbba9frFj0=OqFfea0dXdd9vqai=hGuQ8kuc9pgc9s8qqaq=dirpe0xb9q8qiLsFr0=vr0=vr0dc8meaabaqaciaacaGaaeqabaqabeGadaaakeaacqWGmbatcqWGVbWBcqWGNbWzcuWGmbatgaqcaiabcIcaOiabdIeainaaBaaaleaacqaIXaqmaeqaaOGaeiykaKcaaa@35AD@ was found by grid search on *β*. Under H_0_, *β *was 0. LRT was distributed approximately as χ^2 ^with *N *degrees of freedom. The relative risk of animals having inherited one allele from the parent in question versus animals having inherited the other allele was eβQe−βQ
 MathType@MTEF@5@5@+=feaafiart1ev1aaatCvAUfKttLearuWrP9MDH5MBPbIqV92AaeXatLxBI9gBaebbnrfifHhDYfgasaacH8akY=wiFfYdH8Gipec8Eeeu0xXdbba9frFj0=OqFfea0dXdd9vqai=hGuQ8kuc9pgc9s8qqaq=dirpe0xb9q8qiLsFr0=vr0=vr0dc8meaabaqaciaacaGaaeqabaqabeGadaaakeaadaWcaaqaaiabdwgaLnaaCaaaleqabaacciGae8NSdi2aaSbaaWqaaiabdgfarbqabaaaaaGcbaGaemyzau2aaWbaaSqabeaacqGHsislcqWFYoGydaWgaaadbaGaemyuaefabeaaaaaaaaaa@36A7@. The interval mapping was implemented in a Visual Basic-for-Excel program.

#### QTL mapping using Variance Component analysis on Test-Period Survival (TPS-VC-lin)

TPS was defined as above. A two step variance component method (e.g. [35]) was used. The two steps were:

1. For each putative QTL position on the chromosome segment, calculate the (co-) variance matrix associated with the QTL. This matrix is also called the G or IBD (identical by descent) matrix, and has elements *ij *= Prob(QTL alleles *i *and *j *are IBD). We used the LOKI package [36] to calculate the IBD matrix from the marker information.

2. For each putative QTL position in step 1, construct a model to estimate QTL variance and other parameters, then test for the presence of a QTL.

The model was *s*_*i *_= *μ*****+ *u*_*i *_+ *v*_*ip *_+ *v*_*im *_+ *e*_*i *_where *s*_*i *_is the phenotype of animal i, 0 for affected and 1 for resistant; *μ *is the overall mean; *u*_*i *_is the polygenic effect for animal *i*; *v*_*ip *_is the effect of the paternal QTL allele for animal *i*; *v*_*im *_is the effect of the maternal allele of animal *i*; and *e*_*i *_is a random residual. The random effects *u*, *v*, and *e *are assumed to be distributed as follows: *u *~ N(0, σ_u_^2^**A**), *v *~ N(0, σ_v_^2^**G**), *e *~ N(0, σ_e_^2^**I**), where σ_u_^2^, σ_v_^2^, and σ_e_^2 ^are the polygenic variance, the additive QTL variance of one allele, and the residual variance, respectively. **A **is the additive genetic relationship matrix, **G **is the IBD matrix described above. Parameters σ_u_^2^, σ_v_^2^, and σ_e_^2 ^were estimated using the ASREML statistical package [37], which also calculated the likelihood of the above model. The LRT test statistic was calculated as twice the difference between the likelihoods of the model fitting the QTL and without fitting the QTL (without *v*_*ip *_and *v*_*im*_). LRT has approximately a χ^2 ^distribution with one degree of freedom. Analysis points were at mid-marker values.

#### QTL mapping using Variance Component analysis on Test-Period Survival with logit link (TPS-VC-logit)

This method is very similar to the method above, but accounts for the binary nature of the *s*_*i *_data in Step 2 of the VCs analysis, by using a Generalised Linear Model (GLM) with the logit link function [37], i.e. *Logit*(*s*_*i*_) = *μ *+ *w*_*i *_+ *u*_*i *_+ *v*_*ip *_+ *v*_*im*_. The goodness-of-fit of generalised linear models is measured by their Deviance [38], and a Deviance Ratio Test-statistic (DRT) was calculated as the difference in deviance between a model fitting the QTL and a model without fitting the QTL. The analysis was performed by ASREML [37], which also calculated the deviance. DRT has also approximately a χ^2 ^distribution with one degree of freedom.

#### QTL mapping using Variance Component analysis on Test-Day Survival (TDS-VC-lin)

Following [16], Survival scores (*S*_*ij*_) were given for each animal *i *and day *j *so that *S*_*ij *_= 1 if the fish survived day *j*, *S*_*ij *_= 0 if the fish died at day j, *S*_*ij *_='missing' if the fish was not alive on day *j*, and thus could not show whether it would survive day j or not. The 2-step variance component mapping approach was used also here, where the model used in step 2 was: *S*_*ij *_= *μ*****+ *day*_*j *_+ *u*_*i *_+ *v*_*ip *_+ *v*_*im *_+ *e*ij, where the fixed effect *day*_*j *_accounts for the differences in survival probabilities between days. The likelihood ratio test was calculated as above.

#### QTL mapping using Variance Component analysis on Test-Day Survival with logit link (TDS-VC-logit)

This method is very similar to TDS-lin, but the binary nature of the data is accounted for by a GLM using the logit link function, i.e. the model is: *Logit*(*S*_*ij*_) = *μ*****+ *w*_*i *_+ *day*_*j *_+ *u*_*i *_+ *v*_*ip *_+ *v*_*im*_. The Deviance Ratio Test was calculated as for the TPS-logit model.

## Authors' contributions

TM coordinated and contributed to laboratory work, did data analysis except for VC analysis, and drafted the manuscript with contributions from AKS, THEM, and BH. AKS contributed on VC analysis, was project leader, and took part in writing the paper. BH contributed on VC analysis and in writing the paper. SL provided laboratory facilities, took part in planning of the study and was supervisor of TM. HM did most of the laboratory work. THEM advised and oversaw data analysis and took part in writing the paper. All authors read and approved the manuscript.
